# Sensitivity of SARS-CoV-2 towards Alcohols: Potential for Alcohol-Related Toxicity in Humans

**DOI:** 10.3390/life11121334

**Published:** 2021-12-03

**Authors:** Debasish Basak, Subrata Deb

**Affiliations:** Department of Pharmaceutical Sciences, College of Pharmacy, Larkin University, Miami, FL 33169, USA; DBasak@ULarkin.org

**Keywords:** SARS-CoV-2, COVID-19, alcohol-based hand sanitizers, ethanol, isopropanol, dermal/pulmonary toxicity

## Abstract

Severe acute respiratory syndrome coronavirus 2 (SARS-CoV-2) is the causative organism that is highly contagious and has been responsible for more than 240 million cases and 5 million deaths worldwide. Using masks, soap-based hand washing, and maintaining social distancing are some of the common methods to prevent the spread of the virus. In the absence of any preventive medications, from the outset of pandemic, alcohol-based hand sanitizers (ABHS) have been one of the first-line measures to control transmission of Coronavirus Disease 2019 (COVID-19). The purpose of this narrative review is to evaluate the sensitivity of SARS-CoV-2 towards ABHS and understand their potential adverse effects on humans. Ethanol and isopropanol have been the most commonly used alcohols in ABHS (e.g., gel, solution, spray, wipes, or foam) with alcohol in the range of 70–85% *v*/*v* in World Health Organization or Food and Drug Administration-approved ABHS. The denaturation of proteins around the envelope of SARS-CoV-2 positive sense single-stranded RNA virus is the major mechanism of action of ABHS. Due to frequent use of high-percentage alcohol-containing ABHS over an extended period of time, the oral, dermal, or pulmonary absorption is a possibility. In addition to the systemic toxicity, topical adverse effects such as contact dermatitis and atopic dermatitis are plausible and have been reported during COVID-19. ABHS appear to be effective in controlling the transmission of SARS-CoV-2 with the concern of oral, dermal, or pulmonary absorption.

## 1. Introduction

Coronavirus Disease 2019 (COVID-19), which was first reported from Wuhan, China in December 2019, has inflicted major public health and economic disasters throughout the world. On 11 March 2020, COVID-19 was declared a pandemic by World Health Organization (WHO) [1]. Over the last one year, the understanding of mode of transmission of severe acute respiratory syndrome coronavirus 2 (SARS-CoV-2), the causative organism of COVID-19, has gone through some transformation. It is commonly recognized that direct physical contact and respiratory droplets are the primary modes of transmission among humans. Due to its highly contagious nature, SARS-CoV-2 has been found to cause community spread with ease [2]. Following exposure to SARS-CoV-2, the symptoms can start appearing from second day and continue up to 14 days on average. The typical time to experience symptoms is four to five days [3,4]. The prototype symptoms of COVID-19 include fever, chills, shortness of breath to respiratory distress, and occasionally, impaired taste and smell. The ‘cytokine storm’ or formation and release of inflammatory proteins such as interleukins, tumor necrosis factor α is considered to be one of the hallmarks of COVID-19, especially in severe cases [3]. Due to the extraordinary transmission pattern and severity of this viral infection, as of 27 November 2021, WHO has reported 259,502,031 confirmed cases and 5,183,003 deaths [5]. 

At the onset of the pandemic, the society and medical community went through significant improvisation of strategies to manage the spread and treatment. Because of the nascent nature of COVID-19, there was no approved medications available for prevention and/or treatment. Till now, remdesivir is United States Food and Drug Administration (FDA)-approved only agent for COVID-19 with limited documented efficacy [6]. Apart from remdesivir, bamlanivimab and sotrovimab are two other drugs approved by Health Canada for COVID-19 treatment [7]. In the European Union (EU), remdesivir, regdanvimab, and casirivimab/imdevimab are authorized for the same purpose [8]. Considering the unavailability of effective drugs and scarcity of vaccines against this lethal virus, prevention of transmission using alcohol-based formulations has been the primary strategy [9]. Prevention strategies recommended by the health agencies such as WHO and Centers for Disease Control and Prevention (CDC) early in the pandemic have been highly useful. These measures included social distancing, use of masks, and frequent application of ABHS [1,10]. Despite virucidal property of ABHS towards SARS-CoV-2, the active ingredient alcohols, such as ethanol, isopropanol, in ABHS may pose toxicity to both human health and environment. These toxicities potentially occur through dermal absorption, inhalation, and ingestion following recurrent use. 

The available reports demonstrated that ethanol is more effective against hydrophilic viruses such as rotavirus, human immunodeficiency virus (HIV), and coronaviruses, while isopropanol is superior against lipophilic viruses such as poliovirus and hepatitis A virus [11,12]. Although ABHS are central to the prevention of SARS-CoV-2, their indiscriminate use, and the potential to cause toxicities in humans as well as flammability-related environmental destruction are major concerns. Hence, understanding the effectiveness of ABHS towards SARS-CoV-2 and evaluating the adverse effects that are associated with the use of ABHS are critical. The goal of this narrative review is to appraise the available information on the sensitivity of SARS-CoV-2 towards alcohol-based formulations and highlight the potential adverse effects of ABHS on humans. Specifically, the current work highlighted the general characteristics of SARS-CoV-2, sensitivity of ABHS towards SARS-Cov-2, absorption of alcohol from ABHS, and their related toxicities in humans. The dual effects of ABHS in fighting COVID-19 transmission and potential exposure of humans to alcohol-related toxicities will be appreciated through this study. 

## 2. Methods

**Figure 1:**
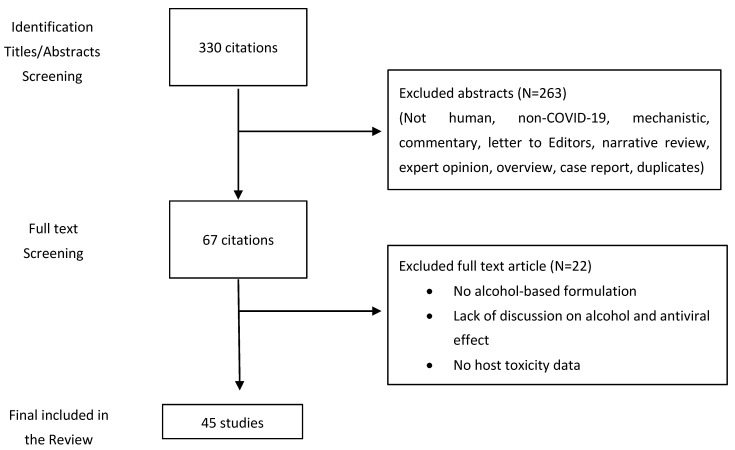
Flow chart of literature search and selection process.

The literature for this narrative review was obtained through a keyword-based targeted search of different electronic databases until 26 November 2021 (Figure 1). Combinations of different keywords were used to acquire relevant articles: “COVID-19”, “coronavirus disease 2019”, “SARS-CoV-2”, “severe acute respiratory syndrome coronavirus 2”, “2019-nCOV”, “2019 novel coronavirus”, “ethanol”, “propanol”, “isopropyl alcohol”, “isopropanol”, “alcohol-based hand sanitizers”, “toxicity”, “dermal”, “inhalation”, and “pulmonary”. We searched PubMed, Medline, and Google Scholar electronic databases. In the first step, the titles and abstracts were analyzed, followed by evaluation of the full text. Original research, government health agency databases, and case studies were included in this review. Studies were excluded if they were not on humans, non-COVID-19, mechanistic, commentary, letter to Editors, narrative review, expert opinion, overview, case report, or duplicates. Both the authors independently performed the literature search and evaluation of articles and reconciled the resources. The present work only includes articles that were published in English language.

## 3. Characteristics of SARS-Cov-2 and Other Coronaviruses

The coronaviruses that belong to the Coronaviridae family are a class of positive sense single-stranded RNA viruses surrounded by envelopes [13]. They are categorized into four groups based on genera and sera: α, β, γ, and δ-coronaviruses. There are seven types of human coronaviruses that are classified under α and β genera only. Among them HCoVNL63 and HCoV-229E fall within α genus and HCoV-HKU1, HCoV-OC43, severe acute respiratory syndrome coronavirus (SARS-CoV), Middle East respiratory syndrome-related coronavirus (MERS-CoV), and the novel SARS-CoV-2 are under the category of β genus. These viruses predominantly cause common cold and respiratory illnesses in humans [14]. On the contrary, γ and δ-coronaviruses mainly trigger avian infectious diseases [15,16].

Like other coronaviruses, SARS-CoV-2 harbors an enveloped positive sense single-stranded RNA genome that is about 30 kb. The genome comprises of a polyprotein ORF1a/b that upon proteolytic cleavage generates 16 non-structural proteins, four major structural proteins, and nine accessory protein ORFs (3a, 3b, 6, 7a, 7b, 8, 9b, 9c, and 10) [17,18,19]. The structural proteins include spike (S), envelope (E), membrane protein (M), and nucleoprotein (N) [20,21]. The spike S protein is a glycoprotein that plays a central role in binding to receptors and is critical for the infective capacity [22]. S protein can be further cleaved by host proteases into an N-terminal S1 subunit and a membrane-bound C-terminal S2 domain. After the virus is internalized into the host endosome through the attachment of S1 subunit to the receptor, the S protein undergoes a conformational change followed by a cathepsin CTSL-mediated cleavage. This leads to the emergence of S2 domain that facilitates fusion of the virion and cellular membranes. The S protein was also reported to have a furin-like cleavage site that contributes to the zoonotic infection of the virus [23]. The E and M proteins are embedded within the S proteins in the virus envelope [24,25].

Genome-wide phylogenetic analysis demonstrates that SARS-CoV-2 is a novel betacoronavirus that is distinct from two other closely related coronaviruses, SARS-CoV and MERS-COV [26]. The SARS-COV-2 shows roughly 79% and 50% sequence identity with SARS-CoV and MERS-CoV, respectively [27]. In the case of structural genes, SARS-CoV-2 exhibits roughly 90% amino acid identity with SARS-CoV except for the S gene [28,29]. On the other hand, SARS-CoV-2 displays more than 85% amino acid sequence identity with SARS-CoV for non-structural proteins. A total of 16 non-structural proteins have been reported in SARS-CoV-2 that are obtained by proteolytic cleavage of a large polyprotein (pp1ab) [17]. The S protein in SARS-CoV-2 (1273 amino acids) is larger than that of SARS-CoV (1255 amino acids). It shares nearly 77% amino acid sequence identity with human SARS-CoV [30]. Moreover, its receptor-binding domain (RBD) demonstrates 73% amino acid identity with SARS-CoV. The S protein is subdivided into S1 and S2 subunits that are linked by four amino acid residues (PRRA) that create the cleavage site which is cleaved by furin and other proteases [23,31]. Harboring of S1-S2 cleavage site is a unique genomic feature of SARS-CoV-2 since others are devoid of such site. A recent report demonstrated that furin-cleavage site is associated with a diminished stability of SARS-CoV-2 S protein, and it enables the virus to assume a conformational adaption that is critical for the binding of the S protein to the Angiotensin Converting Enzyme 2 (ACE2) receptor [31]. However, it is yet to be explored whether the presence of the furin-like cleavage site results in a gain of function that ultimately facilitates the greater transmissibility of SARS-CoV-2 compared with SARS-CoV. SARS-CoV-2 also shares about 88% identity with two bat-derived SARS-like coronaviruses (bat-SL-CoVZC45 and bat-SL-CoVZXC21) [28]. In terms of target receptor, both SARS-COV-2 and SARS-COV interact with ACE2 while MERS-COV interacts with dipeptidyl peptidase 4 (DPP4) also known as CD26 [13,32]. All these coronaviruses transmit through cough droplets and contact with infected individuals.

Although both SARS-CoV and SARS-CoV-2 possess similar receptor-binding domain structures, their amino acids differ at some major residues. These include the absence of 8a protein and variation in the number of amino acids in 8b and 3c proteins in SARS-CoV-2 [28]. Moreover, ORF8 gene of SARS-CoV-2 encodes a protein that shares only 40% amino acid identity with the ORF8 of SARS-CoV [17]. The available reports show distinguishable clinical features as well. For example, the patients infected with MERS and SARS are reported to develop respiratory distress and renal failure in later stages [33,34]. However, in SARS-CoV-2 infection pneumonia is the most prevalent manifestation along with other common symptoms such as fever, cough, and dyspnea [33].

## 4. Alcohol-Based Formulations of Hand Sanitizers

Though the use of alcohol as an antiseptic agent goes back to late 1800s or earlier, ABHS have been used in medical and home settings for about 50 years now [35]. During the ongoing COVID-19 pandemic there have been unprecedent increase in the use of ABHS in all age groups and professions. In the absence of any effective medication for prevention and treatment of COVID-19, ABHS have been one of the major first-line strategies to mitigate the spread of SARS-CoV-2 [1,10]. The formulations of ABHS typically contain different combinations of single or mix of alcohol (ethanol, isopropanol, n-propanol), hydrogen peroxide, humectant (glycerin), and water. In the WHO- and FDA-recommended formulations, the final concentration of alcohol varies between 70–85% *v*/*v* with hydrogen peroxide and glycerol concentrations are fixed at 0.125% and 1.45% *v*/*v*, respectively [36,37,38]. Currently available ABHS are either gel, solution, spray, wipes, or foam with gels and foams being the most commonly available products [36,39]. These ABHS differ among their ability to interact with the skin, contact time, and handling of the products. For example, foams use bis-polyethyleneglycol 12-dimethicone (bis-PEG12-dimethicone) as the foaming agent and has relatively quick drying time [36]. In comparison, gels are sticky in nature but give good contact with the skin to facilitate inhibitory effects on SARS-CoV-2. Solution-based ABHS can have their own advantages such as less contact time and faster drying time and disadvantages such as less stickiness leading to questionable interaction duration with SARS-CoV-2 [39]. Spray types of ABHS mediate direct contact of the product with the skin by triggering steam of alcoholic solution. However, they have the potential of inflammability and over spraying. Wipes are also easy to use, and they can eliminate the contamination possibility. However, due to extended storage wipes may lose their virucidal capacity over time [40]. 

At the onset of pandemic, several health and pharmaceutical agencies such as WHO, FDA, and United States Pharmacopeia (USP) have released policies for compounding of ABHS to address the shortage of hand sanitizers during this public health emergency [37,38,41]. According to FDA guidelines, ABHS ingredients should possess appropriate quality. This includes at least 94.9% pure ethanol, USP-grade isopropanol, food chemical codex (FCC) or USP-grade glycerin, and boiled water/purified water equivalent grade [38]. In addition, food grade alcohol manufactured by fermentation and distillation as well as fuel or technical grade alcohol may also be used if they meet the USP or FCC standards and the impurity levels are within the indicated limits [38]. Thus, ABHS can have a vast range of quality and effectiveness, and due diligence is needed by the user to ensure that the product conforms to the health agency guidelines and is effective to carry out the preventive actions against SARS-CoV-2 transmission. 

## 5. Effects of Different Alcohols on Coronavirus Family

Although initially the virucidal effectiveness of different alcohol-based formulations was controversial, gradually it was unequivocally accepted that enveloped viruses such as coronaviruses can be inactivated by alcohol with ease. Indeed, several strains of coronavirus family including SARS, MERS or HCoV are highly susceptible to 62 to 71% ethanol that can inactivate them in less than one minute. On the contrary, two other biocidal agents, namely 0.02% chlorhexidine digluconate and 0.05%–0.2% benzalkonium chloride did not render much benefit [42]. Siddharta et al. (2017) demonstrated that a 30% concentration of WHO formulation II [75% isopropanol (*w*/*w*)] alleviated bovine coronavirus (BCoV) infectivity; however, at least a 40% concentration of WHO formulation I [85% ethanol (*v*/*v*)] was essential to produce the similar effect [43]. Several studies have implicated alcohol-based formulations to be the agents of choice to fight enveloped viruses including SARS-CoV-2 [43,44]. Kratzel et al. (2020) revealed a similar observation where they found that 80% ethanol and 75% isopropanol inactivated the SARS-CoV-2 strain. Moreover, they also reported that commercially procured ethanol and isopropanol were able to inactivate SARS-CoV-2 in 30 s [37]. A very recent study conducted by Leslie et al. (2021) also lends support to these findings where they demonstrated that commercially available hand sanitizer gel and foam, both containing 70% ethanol (*v*/*v*), can robustly inactivate SARS-CoV-2 [45]. This finding was remarkable since this study employed commercially formulated ABHS marketed in the US for the very first time. Table 1 summarizes the impact of alcohol against various strains of coronaviruses.

**Figure 2:**
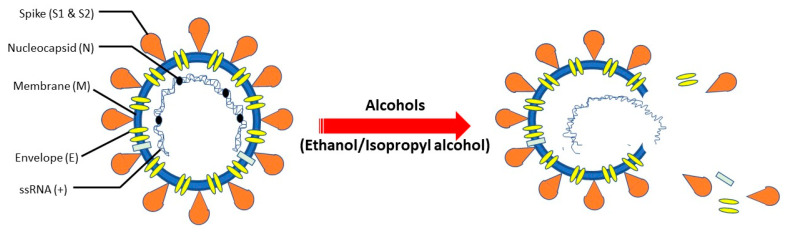
Potential Antiseptic Mechanism of Action of Alcohols on SARS-Cov-2. Alcohols (ethanol/isopropanol) lyse the envelope of SARS-CoV-2 virus and release the internal content which leads to the destruction of the virus. Adapted from [36,54,61].

Denaturation of proteins is conceived to be the major mechanism through which alcohols render their activities against viruses and other microorganisms [52]. Water is indispensable for protein denaturation and that is why absolute alcohol is less effective against the microbes compared to a mixture of alcohol and water [53]. The existing evidence suggests that alcohols can inactivate the lipophilic, enveloped viruses better compared to the non-enveloped viruses [36]. In fact, the envelope is sensitive to lipid solvents and therefore, when the capsid is deprived of the envelope, it may not be able to attach to or interact with the cell surface receptors. Since coronaviruses are lipophilic, enveloped viruses they can be rapidly inactivated by alcohols (e.g., ethanol, isopropanol) [42]. These viruses assume the envelope from the host cells during the budding stage of their life cycle and the envelope is composed of a lipid bilayer. Hence, it is highly likely that even after mutations these coronaviruses will still have the lipid bilayer that can be disinfected by alcohol treatment. Due to the amphiphilic nature of alcohols, they can easily interact with the viral envelope where they alter the membrane fluidity [54]. The polar oxygen moieties strengthen the affinity of the membrane for water and simultaneously diminish the lipophilic interactions between the non-polar residues. In this way, alcohols destabilize and denature the viral proteins and elicit their destruction [55]. In a recent study, Das et al. (2021) employed molecular dynamics simulation where they found that ethanol triggered disintegration of the lipid bilayer and dislocation of the envelope (E)-protein from the membrane environment [56]. Alcohol-mediated lysis of the viral envelope that leads to the release of the internal content is shown in Figure 2. In addition, there are plausible mechanisms of alcohol as virucidal including pH-dependent inactivation, divalent metal ions, and oxidative stress for non-enveloped viruses [57,58,59]. It is worth mentioning that a study reported a greater amplification in virucidal activity against the enveloped coronaviruses with 75% isopropanol compared to the 85% ethanol-based formulation [60]. This can be explained by the fact that isopropanol, by virtue of having one more carbon than ethanol is likely to impart superior lipophilicity against the lipophilic coronaviruses [36]. 

## 6. Oral, Dermal, and Pulmonary Absorption of Alcohols and Their Related Toxicities

The mechanisms of absorption, distribution, metabolism, and excretion of alcohols have been known for decades. Briefly, alcohols are absorbed through the oral, inhalation, or dermal routes to different extents. Alcohols are well distributed across different organs; especially, due to their volatile nature alcohols can easily cross the blood-brain barrier [62,63,64]. Alcohol dehydrogenase (ADH) and aldehyde dehydrogenase (ALDHs) enzymes are responsible for metabolism of different alcohols. Ethanol is converted to acetaldehyde and acetic acid by ADH and ALDH, respectively [62,65]. A small fraction of ethanol is also metabolized by glucuronidation (ethyl glucuronide) and sulfation (ethyl sulfate) pathways [66]. Ethanol remaining after the oxidative and nonoxidative metabolism (<5%) is excreted from the body via urine, sweat, or breath [66]. ABHS contain very high level of ethanol or isopropanol which increases the potential of their absorption through dermal, inhalation, or oral pathways during frequent use over the duration of pandemic. Since very high alcohol-containing ABHS (75–80%) are applied on the hand, there is a potential that alcohol will reach to mouth while consuming food or by inadvertent exposure of hand into the mouth. Most importantly, accidental or intentional ingestion of ABHS can contribute to significant oral absorption of alcohol. Indeed, in US between 2011 and 2014, among 65,293 cases of ABHS-related poisoning, 95% of the cases were oral ingestion in children [67]. The absorption of alcohol follows first-order kinetics signifying the rate of absorption is corresponding to the quantity of alcohol in the stomach [68]. Alcohol can also reach to the systemic circulation through oral mucosa [69,70]. The cardiovascular, central nervous system (CNS), and respiratory effects of isopropanol-based hand sanitizers start demonstrating within an hour of ingestion [71]. The mechanism of gastrointestinal absorption of alcohols involves passive diffusion towards the concentration gradient [65]. Thus, ABHS with high alcohol content have the potential to be absorbed in the systemic circulation through oral route. 

Similarly, both ethanol and isopropanol can enter the systemic circulation, albeit at a lower rate than oral route, through intact skin layers [72,73]. From a pre-COVID-19 study, Turner et al. (2004) reported that blood isopropanol levels increased in nine among the ten individuals tested (range < 0.5–1.8 mg/L) following application of hand rubs six times an hour for four hours [73]. Though the increase in isopropanol was not high enough following dermal absorption to cause intoxication (50 mg/dL), blood acetone (metabolite of isopropanol) level in individuals exposed to isopropanol was not estimated. Similarly, inhalation of vaporized alcohol or droplets produced from the spray or foams have the potential to reach systemic circulation through pulmonary route. It is important to recognize that ethanol can reach to brain quickly through the inhalation route [74,75]. The absorption of alcohol through nasal membrane and alveolar epithelium is faster than the gastrointestinal compartment [76]. ABHS caused an affirmative breathalyzer test following pulmonary absorption of ethanol after a 4-h hospital shift [77]. However, 86 health care workers (18–50 years) did not experience any notable dermal absorption despite multiple usage of ABHS [77]. It is important to note that these studies did not measure the metabolic products of ethanol or isopropanol (e.g., acetaldehyde, acetone) which may have contributed to the lower blood alcohol levels. Additional evidence of dermal or pulmonary absorption of alcohols from ABHS can be interpreted through disulfiram-ethanol reaction as a surrogate marker of alcohol absorption. Disulfiram, which is used to treat alcohol abuse-related problems, can cause unpleasant experiences when the patient has systemic presence of ethanol [78,79]. Ghosh et al. (2021) reported disulfiram-ethanol reaction in about 19% of the participants that were using ABHS. It was postulated that dermal and pulmonary absorption of alcohol contributed to the increased blood alcohol levels and subsequent reaction with disulfiram [79]. Due to high volatility of ethanol and isopropanol along with large dermal surface area or extensive pulmonary vascular structure, the potential to be absorbed and cause systemic effects is high [64,72]. In a recent systematic review published before COVID-19 pandemic started, multiple studies reported increase in breath alcohol content, ethyl glucuronide, and ethyl sulfate following inhalation of alcohol from ABHS [64]. 

Individuals from all age groups have been using ABHS during COVID-19. Intrinsically, alcohols above a certain level have systemic or topical toxic effects in humans [80]. The systemic acute toxic effects of ethanol and isopropanol involve CNS depression, lower respiratory reflex, and nausea [81,82], whereas chronic toxicities include cardiac arrhythmia and hepatic injury [83]. ABHS can contribute to the increase in blood alcohol level-related systemic toxicities and more importantly can cause dermal toxicities such as contact dermatitis and atopic dermatitis. For example, among the 434 healthcare workers surveyed in Hubei, China, 76.6% of the participants indicated conditions similar to dermatitis during COVID-19 [84]. From a mechanistic point of view, skin disorders are a natural progression of extensive use of ABHS where the lipid or emollient contents of the skin is lost and keratin protein is denatured, leading to dry skin, itching, irritation, and eventually clinical diagnosis of dermal disorders [39,85]. ABHS-related skin disorders can also facilitate higher dermal alcohol absorption through cracked skin [80]. Usually, the skin becomes dry very easily after applying ABHS and the healthcare personnel are more susceptible in this regard because of wearing occlusive gloves. Moreover, in cold and dry environment the skin fails to retain moisture making the use of ABHS even more troublesome. Hence, a moisturizer with emollient properties could be beneficial to prevent the skin disruption after using ABHS [86]. Additionally, frequent and extended use of ABHS has posed the danger of antimicrobial resistance to non-viral microbes or virus that are not inherently affected by alcohol [80,85]. 

Though the dermal absorption of alcohol is debatable and a topic of investigation, due to the rapid partition of alcohol in different tissues, the timing of measurement of blood alcohol profile after absorption through skin is critical. Additionally, the unprecedent extensive use of ABHS for such an extended period of time has never been experienced in the modern time. It is important to recognize that despite the focus on dermal or pulmonary absorption from ABHS, there is a huge risk of toxicity from oral exposure or accidental ingestion of ABHS, especially, in the younger people. For example, Pourmand et al. (2021) reported a case where a patient ingested hand sanitizer while staying in the emergency department [87]. Moreover, a recent study by Hohl et al. (2021) reported a greater incidence of ethanol-mediated burns during the pandemic [88]. Indeed, during COVID-19, the reporting of ABHS-related toxicities to poison center has increased in different part of United States [89,90] which also highlights other avenues through which ABHS can cause public health hazards. Table 2 highlights some examples of the adverse effects of different disinfecting agents on human health. 

## 7. Alternatives and Precautions to ABHS

There are certain situations when use of alcohol-based products is not ideal. These include but are not limited to cleaning significantly soiled hands, preventing the spread of non-SARS-CoV-2 infections, and removing excessive bacterial load [95]. Additionally, excessive use of ABHS can demotivate people to use the traditional health hygiene with warm water and soap. At the same time, excessive use of soap and water can result in contact dermatitis and risk of other microbial infections [96,97]. On the contrary, well-formulated alcohol-based sanitizers are devoid of unwanted skin irritation by virtue of having emollients and because of their better compliance, they are indeed preferred to soap and water. Additionally, developing habits of not inserting the ABHS-exposed hands into the eyes, mouth, or nose will minimize alcohol-related toxicities. 

There are certain alternatives to using ABHS. For example, the CDC recommends washing hands with soap and water for at least 20 s to reduce the possibility of SARS-CoV-2 transmission [98]. Moreover, hand wash is a common hygiene practice to remove other microorganisms from the hands [99]. Hence, use of soap is by far the best alternative to use of ABHS. To avoid nasal inhalation and oral ingestion, ABHS should be applied away from the face. Specifically, the hands should be kept away from the face or body and ABHS should be applied gently on the hands, rubbed, and let the hands dry. Using good quality masks such as surgical, FFP2 or N95, and FFP3 masks and face shield are very rational alternative to prevent the spread of the virus [100,101,102]. Furthermore, maintaining proper social distancing and minimize touching high-traffic surfaces are also critical as alternatives to less frequent use of ABHS [103]. It is advised to identify a product that is enriched with humectant to promote hydration (e.g., glycerin) and emollient (e.g., aloe vera) which will minimize skin irritation from ABHS. Water-based antiseptic lotions with benzethonium chloride could be employed to minimize dermal toxicities since this exerts antiviral activity and minimizes flammability [104]. Thus, simultaneous exercise of different preventive measures will facilitate less frequent use of ABHS and use of low-toxicity FDA-approved products will minimize alcohol-related toxicity issues.

## 8. Conclusions

In the absence of targeted antiviral medications, ABHS have been one of the frontline measures in the first year of COVID-19 pandemic. SARS-CoV-2 which is an enveloped positive sense single-stranded RNA is susceptible to damage by alcohol [105]. This principle has been exploited by ABHS to denature the proteins surrounding the envelope which eventually blocks the capsid to attach to the host. Though variable concentrations of alcohol can be found in ABHS, typically 70–85% ethanol or isopropanol is the most common during COVID-19. Available reports suggest that similar to other coronavirus family members, SARS-CoV-2 can be neutralized by ABHS with alcohol content of ≥70% *v*/*v* [106]. Although the effectiveness of ABHS against SARS-CoV-2 appears to be convincing, there are reasonable concerns of oral, dermal, or pulmonary absorption, leading to potential of systemic and/or dermal toxicities. In addition, accidental ingestion of ABHS by younger population has also posed some challenges to the society. Exposure to substandard ABHS containing contraindicated methanol, toxic impurities, or with lower percentage of ethanol or isopropanol, is also an issue that needed attention during this pandemic. Overall, along with other measures, ABHS appear to be effective in controlling the transmission of SARS-CoV-2 with the concern of oral, dermal, or pulmonary absorption and exposure to substandard ABHS. Appropriate education on ABHS use, potential adverse effects, alternatives, and precautions will increase the public health benefits of ABHS. 

## Figures and Tables

**Table 1:** Summary of Alcohol Sensitivity to Different Coronaviruses.

Type	Surface	Survival Time	Virucide	Exposure Duration	Decrease in Infectivity (log10)	Reference
SARS-COV-2	NR	NR	80% ethanol	30 s	≥3.8	[37]
	NR	NR	75% isopropanol	30 s	≥3.8	[37]
MERS-COV	Steel	48 h (20 °C) 8–24 h (30 °C)	80% ethanol	30 s	>4.0	[43,46]
	Plastic	48 h (20 °C) 8–24 h (30 °C)	75% isopropanol	30 s	≥4.0	[43]
SARS-COV	Glass	4 d (RT)	80% ethanol	30 s	≥4.3	[47,48]
	Plastic	≤5 d (22–25 °C)	85% ethanol	30 s	≥5.5	[49,50]
	Wood	4 d (RT)	95% ethanol	30 s	≥5.5	[47,50]
	Paper	4–5 d (RT)	70% isopropanol	30 s	≥3.3	[47,48]
	Gown	2 d (RT)	75% isopropanol	30 s	≥4.0	[43,51]
	Metal	5 d (RT)	100% isopropanol	30 s	≥3.3	[47,48]
	NR	NR	45% isopropanol and 30% 1-propanol	30 s	≥2.8	[48]

RT, Room temperature; NR, Not reported.

**Table 2:** General Mechanisms of Disinfecting Agents and Their Impacts on Human Health.

Agent	Mechanism of Action	Benefits	Drawbacks	Adverse Effects	Reference
Ethanol (>60%)	Denaturation of proteins	Recommended by U.S. FDA against SARS-CoV-2; economical and easy to handle	Disagreeable odor, dryness of skin, possibility of unwanted toxicity in children	Skin: Itching, allergy, dermatitis Liver: Hypocalcemia, hypokalemia, hypomagnesemia, myoglobinuria Others: Vomiting, drowsiness, respiratory arrest, keto acidosis, arrhythmia, cardiac arrest	[42,80,91]
Isopropanol (>70%)	Denaturation of proteins	Recommended by U.S. FDA against SARS-CoV-2; economical and easy to handle	Unpleasant odor, dryness of skin, possibility of unwanted toxicity in children	Skin: Rash, itching, irritation, allergy Others: Myoglobinuria, gastritis, respiratory depression	[42,80,91]
Quaternary ammonium compounds	Enzymatic inactivation; Degradation of cellular proteins	Minimal human toxicity, better tolerability, no bad odor	Less effective in low pH and in the presence of organic substances	Mild irritation	[91,92]
Hydrogen peroxide	Free-radical induced oxidation of cellular components	Relatively less toxic, inexpensive, easy to use	Corrosive	Mild irritation in skin and mucous membrane, vomiting, air embolism	[80,91,93]
Iodine compounds	Degradation of cellular proteins, fatty acids, and nucleotides	Non-corrosive, ease of use	Unpleasant odor, staining, irritation	Rash, itching, local swelling	[91,92]
Chlorine compounds	Halogenation/oxidation of cellular proteins	Effective in low concentration, low-cost	Corrosive, formation of toxic by-products, irritation	Nausea, coughing, shortness of breath, irritation of mucous membrane, stimulation of the upper airways	[91,94]

## Data Availability

Data sharing not applicable.
